# Estimating the costs for implementing a maternity leave cash transfer program for women employed in the informal sector in Brazil and Ghana

**DOI:** 10.1186/s12939-021-01606-z

**Published:** 2022-02-12

**Authors:** Grace Carroll, Mireya Vilar-Compte, Graciela Teruel, Meztli Moncada, David Aban-Tamayo, Heitor Werneck, Ricardo Montes de Moraes, Rafael Pérez-Escamilla

**Affiliations:** 1 Yale School of Public Health, 135 College Street, New Haven, CT 06510 USA; 2 Department of Health, Montclair State University, University Hall 4157, 1 Normal Ave, Montclair, NJ 07043 USA; 3 EQUIDE Research Institute for Equitable Development, Universidad Iberoamericana, Prolongación Paseo de la Reforma 880, Lomas de Santa Fe, 01219 Mexico City, Mexico; 4 Brazilian Health Insurance Regulatory Agency (ANS), Av. Augusto Severo, Rio de Janeiro, 84 Brazil; 5 Brazilian Institute of Geography and Statistics (IBGE), Rio de Janeiro, Brazil; 6 Alberto Coimbra Institute for Graduate Studies and Research in Engineering (COPPE), the Federal University of Rio de Janeiro (UFRJ), Av. Horácio Macedo, 230. Centro de Tecnologia, COPPE/UFRJ, Bloco H, Sala 329. CEP, Rio de Janeiro, RJ 21941-914 Brazil

**Keywords:** breastfeeding, maternity leave, maternity cash transfer, informal sector, costing, Brazil, Ghana

## Abstract

**Background:**

Maternity leave policies are designed to protect gender equality and the health of mothers in the workforce and their children. However, maternity leave schemes are often linked to jobs in the formal sector economy. In low- and middle-income countries a large share of women work in the informal sector, and are not eligible to such benefit. This is worrisome from a social justice and a policy perspective and suggests the need for intervening. Costing the implementation of potential interventions is needed for facilitating informed decisions by policy makers.

**Methods:**

We developed and applied a costing methodology to assess the cost of a maternity leave cash transfer to be operated in the informal sector of the economy in Brazil and Ghana, two countries with very different employment structures and socioeconomic contexts. We conducted sensitivity analysis by modeling different numbers of weeks covered.

**Results:**

In Brazil, the cost of the maternity cash transfer would be between 0.004% and 0.02% of the GDP, while in Ghana it would range between 0.076% and 0.28% of the GDP. The relative cost of rolling out a maternity intervention in Brazil is between 2.2 to 3.2 times the cost in Ghana depending on the benchmark used to assess the welfare measure. The differences in costs between countries was related to differences in labor market structure as well as demographic characteristics.

**Conclusions:**

Findings show how a standard methodology that relies on routinely available information is feasible and could assist policymakers in estimating the costs of supporting a maternity cash transfer for women employed in the informal sector, such intervention is expected to contribute to social justice, gender equity, and health trajectories.

## Background

Maternity leave policies are designed to protect gender equality and the health of mothers in the workforce and their children [1, 2]. Globally, paid maternity leave has gradually become a standard social benefit with more than half (53%) of the countries around the world now adopting the International Labor Organization (ILO) standard of at least 14 weeks of leave [3, 4]. However, women who work in the informal economy, are commonly not covered by formal arrangements [5]. This is worrisome from a social justice and a policy perspective as women make up a disproportionate percentage of employees in the informal sector, especially in low- and middle-income countries. According to data from the ILO, in Southern Asia over 90% of women, 92% in sub-Saharan Africa, and 54.3% in Latin American and the Caribbean are employed in the informal economy [6].

Lack of maternity leave coverage among women working in the informal sector, commonly leads pregnant women to continue working far into their pregnancy and return to work too soon after childbirth, exposing themselves and possibly their children to unnecessary health, nutrition, and developmental risks. This decision is driven by the fact that if they stop working even for a short period of time, they are likely to face increased household income insecurity.

Paid maternity leave could provide the income protection needed to delay the decision to return to work among women in the informal economy. In fact, previous studies with women in the formal economy have found that paid maternity leave is also positively associated with improved mental and physical health of mothers and children [2, 7, 8]. Hence the difference in the level of maternity protection between women employed in the informal sector versus those employed in the formal sector represents a major inequity and human rights violation with major household and social repercussions. Such unequal maternity protection violates the Convention on the Rights of the Child [9] that states the right to life, survival and development (Article 6), as well as the Convention on the Elimination of All Forms of Discrimination Against Women [10], which advocates for a proper understanding of maternity as a social function, and proclaims maternity protection and child-care as essential rights (Article 5).

Maternity leave benefits have also been associated with more optimal breastfeeding practices [2, 11, 12], including exclusive breastfeeding (EBF) for the first 6 months and breastfeeding continuation up to 2 years and beyond. Hence, an important negative consequence associated with the lack of maternity leave benefits is to prevent children, mothers, and society at large from receiving the benefits that breastfeeding offers [13, 14]. Breastfed infants compared to non-breastfed have improved cognitive development, reduced risk of overweight and obesity and fewer childhood illnesses such as gastrointestinal infections and pneumonia [15, 16]. Moreover, breastfeeding reduces the mother's risk of ovarian and breast cancer, type 2 diabetes, and cardiovascular diseases [14, 17]. According to Walters et al [18] globally, close to 600 thousand childhood deaths are attributed to not breastfeeding per year, and the total global economic losses of suboptimal breastfeeding are estimated to be between US$257 billion and US$341 billion annually. Therefore, the lack of maternity protection among informally employed women represents a major health related inequity [14, 19] and an important barrier to optimal development [18].

There is strong justification for developing effective legal and policy frameworks to mandate maternity benefits for women employed in the informal sector who are commonly ineligible for social security benefits including maternity leave. However, expanding access to benefits such as these remains a complex challenge. Although the ideal solution would be to provide equal labor protections to all women regardless of their source of employment, this would require a structural change that is unlikely to happen in the short-term. Such structural change would imply, incorporating workers operating within non-standard employment relationships (i.e. domestic workers, transport workers, trash collectors, etc.), within non-standard workspaces (i.e. streets), and oftentimes socially vulnerable groups such as women; into contributory social protection mandates [20]. This will require long-term labor sector modifications grounded in robust organizational, fiscal, and legal frameworks. Hence, there is an urgent need to propose shorter-term innovative and pragmatic approaches to provide maternity benefits to informally employed women. A policy instrument proposed by the ILO is a maternal cash transfer [3, 21]. Cash transfers have been increasingly adopted as social protection strategies in many low- and middle-income countries (LMICs) [22]. A recent systematic literature review and a realist review concluded that cash transfers can provide a wide range of beneficial health, nutrition, economic, and social benefits (e.g., income sources) to women, children, and other individuals living in households in LMICs [23, 24]. Interestingly, none of the cash transfers included in these reviews specifically included a maternity cash transfer. However, there is indirect evidence that maternity benefits based on a cash transfer approach are likely to work as, for example, non-contributory pensions or those seeking to reduce child labor report a reduction in labor intensity (i.e., the time spent working) [23].

Cash transfer programs can also help foster gender equity as often times the transfers are provided directly to women [23–25]. Empirical evidence from different world regions strongly suggests that interventions such as a maternity cash transfer could help protect households against economic shocks [26]. A maternity cash transfer could have short-term effects, such as increasing the chances that a working woman could stay home with her baby without facing an economic contraction, ensure a basic level of income for informally employed women and their families, and, in turn, benefit from long-term effects including improved health and nutrition for the infant, and human capital development and gender equity, by better addressing the needs of women and girls [25]. As these types of policy instruments are just being considered, there are gaps in understanding how much the maternity cash transfer would cost and how it can be delivered. From a programmatic and policy perspective, this information is crucial for policymakers to understand the feasibility of extending such an intervention as well as to gauge the funds that are necessary to budget towards this end.

A macro-costing framework to estimate the annual cost of a maternity leave cash transfer for informally employed women was recently developed [27] and has been successfully applied to Mexico [27], Indonesia [28], and the Philippines [29]. We aim to expand this body of literature by estimating the cost of implementation of a maternity leave cash transfer in two LMICs with quite contrasting economic, social, and political contexts, Ghana and Brazil. This work is useful and informative because none of the prior costing studies have compared the maternity cash transfer costs in countries with different employment structures and varying regional contexts using one standard costing methodology. Therefore, in this study, we seek to test the adaptability of the costing approach in countries with different informal sector challenges. Currently, neither Brazil nor Ghana have a program or intervention package in place to offer maternity protection to women employed in the informal sector. These countries are different across several domains: economic development (GDP is almost 3 times higher in Brazil than Ghana), geographic region (sub-Saharan Africa, South America), labor market structure (including women participation rate in the informal sector, which is twice as much in Ghana, 83.2%, than in Brazil, 38.2%), fertility rates (higher in Ghana, 3.9, than in Brazil, 1.7), and breastfeeding indicators (larger in Ghana, 52.1%, than in Brazil, 45%) (Table 1).

**Table 1:** Characteristics of the countries: Brazil and Ghana

Variable	Brazil	Ghana
Total Population, no	211,049,527	30,417,856
GDP per capita, PPP$	14652	5413
Informal employment, % ^a^	38.27	83.18
Working-age population, % (15-64 years)	69.74	59.54
Labor force female, %	43.58	46.50
Population of women, no. (%)	107,316,363 (50.85)	15,001,771 (49.32)
Fertility rates, total births per woman	1.73	3.87
Current duration, maternity leave (weeks) for the formal sector^b^	17	12
Exclusive breastfeeding, % of children aged under 6 months^c^	45.0	52.1

Notes: GDP, Gross Domestic Product; PPP$, Purchasing Power Party constant 2017 international dollars.

^a^Informal employment is based on a harmonized measure of the International Labour Organization (ILO), is reported in the World Development indicators 2015 [30].

^b^Data were form ILO 2014 [3].

^c^Data for Ghana was obtained from the World Development Indicators 2014 [30] and for Brazil from the Indicadores de aleitamentomaterno no Brasil, ENANI [31].

Data sources: World Development Indicators 2019 [30] (unless otherwise specified).

## Methods

### Settings

 We estimated the costs for implementing a maternity cash transfer for mothers employed in the informal sector, using nationally representative cross-sectional data, employment, and fertility data, from Brazil and Ghana. The data were comparable across countries thematic wise, but were collected at different times; data were collected in 2015 for Brazil and 2017 for Ghana.

### Costing methodology

 To estimate the annual cost of implementing the maternity cash transfer for informally employed women, we used the methodology proposed by Vilar-Compte et al. [27], which was an adaptation from a costing methodology from the World Bank [32, 33], designed to estimate the financial needs for scaling up nutrition interventions to achieve the World Health Assembly global nutrition objectives.

The costing approach followed in our study is based on the following equation:

$${ML}_y= CT\ast {IC}_y\ast \left(\alpha \ast {Pop}_y\right)+{AdmCost}_y$$

where *ML*_*y*_ is the maternity cash transfer (CT) cost needed for a year of intervention, *CT* is the CT unit cost, *IC*_*y*_ is the number of weeks the *CT* would cover a woman in year *y*, and (*α* ∗ *Pop*_*y*_) is the population of women of reproductive and legal working ages in a given country in year *y* weighted by *α* (probability of having given birth according to women’s demographic characteristics). *AdmCost*_*y*_ refers to the administrative costs in a given year required to operate the intervention.

A key aspect of our costing methodology is that it is based on six clearly defined steps that can be replicated across countries. The methodology requires nationally representative survey data on employment and fertility, as well as demographic data to adequately weight the population size, all of which are commonly available in most countries.

### Application of the costing methodology

 The costs of implementing a maternity CT for informally employed women was estimated in Brazil and Ghana using the six-step methodology (Table 2).

- **Step 1** estimated the number of women of reproductive and legal working ages who reported having a child in the last year (Table 2). This information was needed to compute *α*. Based on these data, women of reproductive age were categorized by demographic subgroups according to their age, marital status, educational attainment, and urban-rural area of residence. Although the objective was to fully harmonize the estimation process for Brazil and Ghana, we also aimed to capture local conditions. Therefore, slight differences in variable categorization occurred between countries due to contextual differences (i.e., the number of categories of educational levels) (Table 2). These differences explain the different number of possible combinations of women’s characteristics in each country. For each of these combinations, the proportion of women who reported giving birth in the previous year was estimated. For example, the proportion of women 16 to 24 years old, single and without education, living in an urban area in Brazil and who reported having a baby in the prior year was 10.3%.
- **Step 2** focused on determining the probability of a woman working in the informal sector having had a baby in the prior year (*α*). This required defining informal employment, which varied between countries (Table 2). Then using the combinations generated in Step 1, employment information was applied to estimate the probability of having had a child in the prior year among informally employed women. This required linking fertility and employment data for each subgroup combination.
- **Step 3** centered on identifying the target population *Pop*_*y*_ (women of reproductive and legal working age in each country) through national population estimates using the World Bank population projections for both countries (Table 2). To compute (*α* ∗ *Pop*_*y*_),the national population of women of reproductive age was then weighted by each of the *α* ′ *s* estimated in Step 2.
- **Step 4** estimated the *CT* amount that could be provided to informally employed women. It was defined through two common welfare measures: the minimum wage and the income poverty line. The minimum wage referred to a “wage floor” and was intended to be sufficient to cover the costs associated with minimum family living expenses. There were different mechanisms in how each country sets such wages. For purposes of the current analysis, we retrieved the minimum wages for Brazil and Ghana from the *WageIndicator* [34] and standardized them to weekly values. Poverty lines were equivalent to thresholds estimating the minimum level of income deemed adequate for a given country or region. We used the World Bank poverty lines that are based on the costs of living for basic food, clothing, and shelter. As the poverty line represents a basic threshold, we also estimated the cost of a CT at two times the poverty line.
  These weekly estimates represented different proxies of the weekly cash transfer (*CT*). For the costing estimations, *CT* were multiplied to the weighted population estimated in prior steps, *CT* ∗ (*α* ∗ *Pop*_*y*_). An important assumption in this step is that the *CT* would be provided to all women working in the informal sector while having an infant. However, different assumptions can be made about coverage, and incremental expansions (Table 2). This could be especially important for countries like Ghana that have a large share of women employed in the informal sector.

**Table 2:** Methodological steps for estimating the annual costs of a maternity cash transfer for informally employed women in Brazil and Ghana

Step	Aim	Data used	Process	Variables input	Notes
1	Compute the probability of a women having a baby in the previous year, given a set of women’s characteristics, needed to compute the value of α in Step 2	Fertility data sources: **• Brazil**: National Household Sample Survey 2015 (PNAD) [44] **• Ghana**: Ghana Living Standard Survey 2017 (GLSS7) [45]	**•** Identify women of reproductive age (16-49 years) **•** Among this subset of women, generate combinations based on: Age Marital status Educational level Locality (based on country level definitions) **•** For each of the combinations, calculate the percentage that had a live birth in the prior year (as a proportion of the total number of women of reproductive age)	Age groups **• Brazil& Ghana**: 16-24; 25-29: 30-34; 35-39; 40-49. Marital status **• Brazil& Ghana**: single; married/living with a man; widow/divorced/separated. Educational level **• Brazil**: no education; kindergarten or incomplete primary; complete primary or incomplete middle; complete middle or incomplete high school; complete high school; higher or any technical career. **• Ghana**: no education; primary or kindergarten; secondary/middle or incomplete high school; complete high school or higher incomplete or technical career; higher complete or more. Locality **• Brazil & Ghana**: rural; urban	Number of combinations: **• Brazil**: 180 **• Ghana**: 150
2	Estimate the probability of a women working in the informal sector having a baby in the prior year (***α***), given a set of women’s characteristics	Fertility and employment data: **• Brazil**: National Household Sample Survey 2015 (PNAD) [44] **• Ghana**: Ghana Living Standard Survey 2017 (GLSS7) [45]	**•** Define informal employment **•** Using the demographic groups generated in step 1, add employment information to estimate the probability of having a baby only among informally employed women	Informal employment **• Brazil**: individuals without a formal contract, including domestic workers, employers and self-employed workers who do not contribute to social security, unpaid workers, as well as workers in production for own consumption and construction for own use. Variables to operationalize: occupation and social security contribution [46] **• Ghana**: individuals who don’t have at least one social benefit (maternity leave, sick leave or holidays) and were without a written or verbal contract [47]. Variables to operationalize: holidays, paid leaves and contract	Employment in the formal and informal sector can vary by each country, national definitions should be prioritized [6]
3	Estimate the population of women of reproductive age weighted by the probability of having a baby in the previous year based on individual characteristics (***α ∗ Pop***_***y***_**)** This step seeks to generate a more realistic estimate the number of women employed in the informal sector who may claim maternity leave in a given year (i.e., target beneficiaries)	Census data: **• Brazil:** 2010 Census (IBGE 2012) **• Ghana:** 2010 Census (GSS 2010) Population projections: **• Brazil**: World Bank 2015 population projections for age group [48] **• Ghana**: World Bank 2017 population projections for age group [48] Employment Data: **• Brazil**: National Household Sample Survey 2015 (PNAD) [44] **• Ghana**: Ghana Living Standard Survey 2017 (GSS 2017) [45]	**•** Identify national estimates of women of reproductive age (16-49 years) currently working in the informal sector for *Pop*_*y*_ **•** Multiply the population by each of the values of α’s generated in step 2	**•** Number of women 16-49 years currently working in the informal sector	While some surveys used in steps 1 and 2 may have expansion factors (e.g., Brazil), we strongly recommend not using them as they were generated for expanding other population subgroups. This may increase the error of any estimated parameter.
4	Estimate the weekly cost (***UC***_***CT***_) of the maternity cash transfer (**CT)** using common welfare measures (i.e., minimum wages, poverty lines).	Minimum wage: **•** For both countries were retrieved from the *WageIndicator* [34]. This information can be retrieved from national offices as well. Poverty lines: **•** For both countries were estimated based on the oncome poverty line from the World Bank [36]	**•** Determine the weekly maternity cash transfer through minimum wages and poverty lines	Cash transfers: **•** Estimations can be performed through different operationalizations of the minimum wage and the poverty line, which may depend on contextual aspects. **•** For purposes of the current analysis the maternity cash transfer was estimated at: the minimum wage the poverty line twice the poverty line	The assumption for the two countries was that maternity cash transfer would be provided to all eligible women in one year, but incremental coverage could be modelled.
5	Determine the number of weeks to be covered, or incremental weekly coverage of the maternity cash transfer **(*IC***_***y***_**)** according to relevant thresholds	International and national organization documents establishing length of maternity leave coverage	**•** Determine the duration of the maternity leave cash transfer program.	For both Brazil and Ghana, the following durations were used for comparing estimates: **• 12 weeks:** current duration of maternity leave in the formal sector in Ghana **• 14 weeks**: duration recommended by the ILO **• 18 weeks**: duration of maternity leave for formal workers currently discussed by key stakeholders in Ghana and approximate current duration of maternity leave in the formal sector in Brazil; extension recommended by the ILO R191. **• 26 weeks:** durations to support EBF	Could include durations established policies for the formal sector
6	Determine the administrative cost of operating the maternity leave cash transfer program **(*AdmCost***_***y***_). Multiply the weekly cost of the maternity CT (*UC*_*CT*_) by incremental coverage **(*IC***_***y***_**)** by the weighted population (***α ∗ Pop***_***y***_) andadd the yearly administrative costs **(*AdmCost***_***y***_) to determine the total annual cost of the maternity cash transfer program (***MatCT***_***y***_).	Administrative costs of programs similar in structure (i.e. one-time subsidy for a specific purpose) or from the same intervention in similar countries: **• Brazil**: the estimated administrative costs for the Mexican maternity cash transfer [27] **• Ghana**: average of the administrative costs of two programs (i.e., LESDEP and NHIS) [49]	Multiply the number of weeks to be covered (***UC***_***CT***_) by (***IC***_***y***_) by (***α ∗ Pop***_***y***_**)**. This will estimate the annual cost of the expansion in the maternity leave coverage. Add the administrative costs to (*α* ∗ *Pop*_*y*_) ∗ *CT* ∗ *IC*_*y*_	Administrative costs: **• Brazil**= 5.6% **• Ghana**=5.8%	This step requires gathering the best locally available data to estimate the administrative costs. Sometimes it can be retrieved from national budgets if they are publicly available [27]. If unavailable, regional data may be used [32].

EBF=exclusive breastfeeding; ILO=International Labour Organization; WRA=women of reproductive age.
- **Step 5** centered on assessing different scenarios according to the number of weeks covered, which is the incremental coverage (IC*)*. We assessed four relevant alternatives: (i) 12 weeks, which was the current number of weeks covered in Ghana for formally employed women, although this threshold was below what it is currently offered to formally employed women in Brazil; (ii) 14 weeks, which was the minimum coverage recommended by the ILO, (iii) 18 weeks which was being considered by stakeholders as a potential extension for formally employed women in Ghana at the time of the study and which coincides with the duration recommended by the ILO R191 [35], and would imply a similar coverage to formally employed women in Brazil, and (iv) 26 weeks that would be consistent with the WHO recommendations regarding EBF for the first six months of life. To estimate *IC*_*y*_ ∗ *CT* ∗ (*α* ∗ *Pop*_*y*_), the *IC* was then multiplied by the weighted population and the *CT*.
- **Step 6** estimated the administrative costs of setting up and managing the maternity CT. The annual administrative costs were computed using other programs as proxies. For Ghana, information was retrieved from the World Bank about the administrative costs of different social programs. Those that had relatively simple administrative structures to deploy resources were selected (i.e., National Health Insurance Scheme, NHIS, and the Local Entrepreneur and Skill Development Program, LESDEP) and their administrative costs were averaged (approx. 5.8%). Other more sophisticated programs, such as the poverty alleviation CT program named Livelihood Empowerment Against Poverty (LEAP), were not considered appropriate for our modeling as they provide benefits to low-income families for several years through a large and complex infrastructure. We assumed that this infrastructure would not be needed for the maternity CT that would be provided as one payment per pregnancy-child. For Brazil, we used the percentage of administrative costs computed by Vilar-Compte et al [27] for the Mexican maternity cash transfer costing estimations (approx. 5.6%). This decision was based on the similarities in administrative costs in both countries for conditional CT programs such as Bolsa Familia and PROSPERA. In addition, both Brazil and Mexico are upper-middle income countries, with high levels of social inequality and similar operational challenges.
*CT* ∗ *IC*_*y*_ ∗ (*α* ∗ *Pop*_*y*_) + *AdmCost*_*y*_ was estimated by adding the administrative costs to the estimations performed in steps 1 to 5.

All cost estimations were conducted with Stata, version 15 (STATA Corp, College Station USA) expressed in US$ and PPP$ using 2018 as a reference year.

## Results

Table 3 presents the characteristics of informally employed women in both countries and the estimated proportions who gave birth in the previous year. The weekly *CT* in Brazil ranged between PPP$43.2 and PPP$106.6 per woman, and in Ghana between PPP$18.9 and PPP$37.8 per woman. For both countries, the CT estimated through the minimum wage was the highest (Table 4).

**Table 3:** Characteristics of women of reproductive age informally employed in Brazil and Ghana

Variable by country	Women informally employed	
	Estimated total % (n)	Estimated % (n) giving birth in previous year
**Brazil**		
*Age, years*		
16 to 24	20.1(3,592)	4.2(151)
25 to 29	14.4(2,562)	4.5(115)
30 to 34	16.9(3,016)	3.8(115)
35 to 39	17.9(3,187)	2.1(67)
40 to 49	30.8(5,493)	0.1(5)
*Education level*		
No education	4.1(739)	1.6(12)
Kindergarten or incomplete primary	10.1(1,802)	2.3(41)
Complete primary or incomplete middle	13.4(2,390)	2.8(7)
Complete middle or incomplete high school	20.5(3,651)	3.0(110)
Complete high school	35.6(6,363)	2.7(172)
Higher or any technical career	10.1(1,802)	2.6(47)
*Marital status*		
Single	35.4(6,324)	1.8(114)
Married/living with a man	56.6(10,109)	3.3(334)
Widow/divorced/ separated	7.9(1,417)	1.7(24)
*Locality*		
Urban	87.6(15,633)	2.6(406)
Rural	12.4(2,217)	2.9(64)
**Ghana**		
*Age, years*		
16 to 24	25.9(2,368)	8.5(201)
25 to 29	15.7(1,431)	16.3(233)
30 to 34	16.4(1,499)	14.1(211)
35 to 39	15.5(1,411)	7.3(103)
40 to 49	26.5(2,418)	2.9(70)
*Education level*		
No education	32.3(2,945)	10.7(315)
Primary or kindergarten	19.9(1,819)	10.2(186)
Secondary/middle or incomplete high school	37.0(3,377)	7.6(257)
Complete high school or higher education incomplete or technical career	10.6(965)	6.4(62)
Higher complete or more	0.2(21)	4.7(1)
*Marital status*		
Single	23.6(2,152)	2.8(60)
Married/living with a man	65.6(5,991)	12.2(731)
Widow/divorced/ separated	10.8(984)	2.8(28)
*Locality*		
Urban	34.7(3,164)	10.6(335)
Rural	65.3(5,963)	5.9(352)

*Notes:* Brazilian estimations were based on PNAD (2015) [44]. Ghanaian estimations were based on GLSS (2017) [45].

**Table 4:** Different operationalization assumptions for maternity cash transfer in Ghana and Brazil based on welfare measures.

	Welfare Reference Measure	Operationalization	Weekly CT	
**Brazil**	Minimum wage	*Full*	US$	*58.3*
			PPP$	*106.6*
	Poverty line	*Full*	US$	*23.6*
			PPP$	*43.2*
	Poverty line	*Two times*	US$	*47.3*
			PPP$	*86.4*
**Ghana**	Minimum wage	*Full*	US$	*11.7*
			PPP$	*32.6*
	Poverty line	*Full*	US$	*6.8*
			PPP$	*18.9*
	Poverty line	*Two times*	US$	*13.5*
			PPP$	*37.8*

CT=cash transfer; PPP=purchasing power parity; US=United States.

Notes: The minimum wage corresponds to 2019 in both countries. Poverty line corresponds to World Bank poverty line recommendations for upper-middle-income countries (PPP5.50 per day in Brazil) and lower-middle-income countries (PPP3.20 per day in Ghana) [36]. Values were reported in 2019 US dollars and 2019 PPPs

The estimated number of eligible women to receive the CT benefit was 291,699 in Brazil and 434,410 in Ghana (Table 5). We computed the total cost of the maternity cash transfer, *MLy* = *CT* ∗ *ICy* ∗ (*α* ∗ *Popy*) + *AdmCosty* considering *IC* of 12, 14, 18, and 26 weeks (Table 5). In Brazil, implementing a maternity cash transfer for 12 weeks would cost between PPP$159 million annually using the poverty line and PPP$393 million annually with the minimum wage, corresponding to PPP$547 to PPP$1,350 per woman (Table 5) per year. In Ghana, implementing a maternity cash transfer for 12 weeks would cost between PPP$104 million annually with the poverty line and PPP$179 million annually with the minimum wage, corresponding to PPP$240 to PPP$414 per woman (Table 5) per year. Extending the weekly duration of the maternity cash transfer would logically increase the costs in both countries (Table 5).

**Table 5:** Estimated costs of an annual maternity cash transfer for women informally employed in Brazil and Ghana at different week duration

Variable	Brazil		Ghana	
**Population of eligible women**	291,699		434,410	
**Annual cost per 12 weeks of MCT**	**Total Cost**	**Cost per women**	**Total Cost**	**Cost per women**
Minimum wage US$	215,430,093	739	64,374,448	148
Minimum wage PPP$	393,709,016	1,350	179,716,519	414
Poverty line US$	87,357,703	299	37,326,468	86
Poverty line PPP$	159,650,467	547	104,205,673	240
Twice the poverty line US$	174,715,407	599	74,652,936	172
Twice the poverty line PPP$	319,300,935	1,095	208,411,347	480
**Annual cost per 14 weeks of MCT**				
Minimum wage US$	251,335,094	862	75,103,525	173
Minimum wage PPP$	459,327,163	1,575	209,669,277	483
Poverty line US$	101,917,323	349	43,547,547	100
Poverty line PPP$	186,258,883	639	121,573,291	280
Twice the poverty line US$	203,834,646	699	87,095,094	200
Twice the poverty line PPP$	372,517,766	1,277	243,146,581	560
**Annual cost per 18 weeks of MCT**				
Minimum wage US$	323,145,120	1,108	96,561,673	222
Minimum wage PPP$	590,563,489	2,025	269,574,779	621
Poverty line US$	131,036,559	449	55,989,704	129
Poverty line PPP$	239,475,716	821	156,308,517	360
Twice the poverty line US$	262,073,119	898	111,979,409	258
Twice the poverty line PPP$	478,951,432	1,642	312,617,034	720
**Annual cost per 26 weeks of MCT**				
Minimum wage US$	466,765,205	1,600	139,477,979	321
Minimum wage PPP$	853,036,213	2,924	389,385,808	896
Poverty line US$	189,275,038	649	80,874,015	186
Poverty line PPP$	345,909,370	1,186	225,778,962	520
Twice the poverty line US$	378,550,076	1,298	161,748,030	372
Twice the poverty line PPP$	691,818,740	2,372	451,557,923	1039

CT=cash transfer; PPP=purchasing power parity; US=United States. *Notes:* Brazilian estimations were based on PNAD (2015) [44], World Bank population projections for women 16-49 years in Brazil from 2010-2015 [48]. Ghanaian estimations were based on GLSS (2017) [45] and World Bank population projections for women between 16-49 years from 2010-2017 [48].

In Brazil, the cost of the maternity cash transfer would be between 0.004% and 0.02% of the GDP, while in Ghana it would range between 0.076% and 0.28% of the GDP.

## Discussion

Informally employed mothers generally face a constellation of vulnerabilities linked to lack of labor and social security protection. Lack of maternity protection violates child’s health and development rights, as well as gender equity promoting international agreements that prompt the need for policy action. On light of this need, our study estimated different scenarios for the cost of implementing a maternity cash transfer for women employed in the informal sector in Brazil and Ghana.

Brazil and Ghana are middle-income countries that are similar with regards to not granting protection to mothers working in the informal sector, but are different with regards to their labor structures and demographic characteristics. For example, despite Brazil having a total greater population size, the estimated number of potential beneficiaries in Ghana was greater due to a much larger proportion of women working in the informal economy than in Brazil. Additionally, the fertility rate was different in both countries, a fact that also contributed to a greater estimated number of potential beneficiaries in Ghana. This highlights the importance of weighting the population by *α* (i.e. probability of a woman working in the informal sector having had a baby in the prior year).

Although the estimated number of potential beneficiaries in Ghana was greater, the total cost per mother estimates in Brazil were still higher. This resulted from differences in welfare measures between Ghana, a lower-middle income country, and Brazil, an upper-middle income country. However, differences in the size of the economies, this implied that the annual cost of implementing a maternity cash transfer would be lower as a share GDP in Brazil than in Ghana. This highlights two aspects: the importance of considering the context of countries, and the adaptability of the methodology, which is sensitive to such variations.

Implementing a maternity cash transfer will imply the interaction of different stakeholders, institutions, and contextual factors [37]. Cost analyses will help stakeholders understand and advocate for the necessary budgetary resources to implement and sustain such health and equity promoting intervention for women and children [37, 38]. To our knowledge, maternity cash transfers have not yet been implemented but our estimates from different countries indicate that its annual cost would imply a low share of the GDP, not exceeding 0.5% in Indonesia [28] and Ghana, and less than 0.1% in the Philippines [29], Mexico [27] and Brazil. Such shares reflect lower investments in relationship to the estimated costs of not breastfeeding [18].

In addition, prior literature has documented that paid maternity leave schemes have positive impacts on maternal and child social, developmental, and health benefits [39, 40]. While this has been documented among women working in the formal sector, it is plausible that a parallel impact could be achieved in women working in the informal economy. This is a hypothesis that will need to be tested, as the living conditions and benefits of formally employed women may arise from a combination of social, economic and environmental factors, and not just due to the maternity benefit. Hence, measuring the effectiveness of maternity cash transfers for informally employed women will be fundamental to address the social return of the investments computed through the costing methodology. This is a key area for future research that should target the benefits on the mothers’ health and employment trajectories as well as the health and developmental outcomes of infants. In addition, these interventions should be understood from a social justice and gender equity perspective [41].

The current research has some limitations. First, despite our efforts to standardize the costing method, there were variations in the national-level surveys, such as time periods of data collection and structure of surveys. Despite such differences in the data sources in each country, we were able to estimate the relevant parameters. A hindrance linked to the standardization was in terms of differences between countries in the definition of some variables, like education, that led to the different categorization of variables. This does not affect the application of the methodology to estimate parameters that are applicable and valid to each context. Second, the administrative costs were calculated from analogous programs – in Ghana from other subsidies with a potential similar structure, and in Brazil by imputing the percentage of the administrative costs estimated for a similar country [27]. This is an area that will require further research if such cash transfers start to get implemented. The administrative costs will need to include start-up costs, fixed as well as variable costs. A third limitation of the study is that it only computes the initial year of the intervention. This decision was based on the relevance identified in prior work about profiling this intervention as something feasible for policymakers [27–29]. If maternity cash transfers start becoming a reality, more robust long-term cost analyses will be needed.

It is fundamental to acknowledge that while maternity leave protection is a key policy to promote and support working mothers and their babies (i.e., employment trajectories, empowerment, breastfeeding choices, nurturing care), other areas of intervention should also be addressed to ascertain that informally employed women have fairer opportunities such as workplace policies, and childcare amongst others. It is also relevant to acknowledge that some unintended effects of cash transfers have been reported in prior literature, including increases in the probability of childbirth and pregnancy when the amount transferred is a function of the number of children [42].

In the context of the COVID pandemic, interventions like the proposed maternity cash transfer should be part of a package of urgent policy actions. The pandemic has aggravated gender inequities specially among informally employed women who had increased childcare responsibilities, financial fragility, and limited remote work opportunities. In fact, a UN-Women report has stressed that there is urgent need for actions to be taken to increase the social protection for informally employed women through policies such as cash transfers [43].

## Conclusions

Findings show how a standard methodology that relies on routinely available information is feasible and could assist policymakers in estimating the costs of supporting a maternity cash transfer for women employed in the informal sector. Supportive labor market interventions are fundamental for informally employed women, especially in LMICs, as they promote gender equity, and social rights for mothers and children. In addition, from the standpoint of infant and young child feeding nutrition, maternity protection interventions highlight that breastfeeding is a collective social responsibility that requires actions and investments.

## Data Availability

Data analyzed was publicly available, the specific STATA Code is available upon request

## Abbreviations

CT: Cash Transfer
EBF: Exclusive Breastfeeding
GDP: Gross Domestic Product
IC: Incremental Coverage
ILO: International Labor Organization
LEAP: Livelihood Empowerment Against Poverty
LESDEP: Local Entrepreneur and Skill Development Program
LMICS: Low- And Middle-Income Countries
NHIS: National Health Insurance Scheme
WRA: Women of Reproductive Age
